# Zopiclone versus placebo for short-term treatment of insomnia in patients with advanced cancer—a double-blind, randomized placebo-controlled clinical multicenter phase IV trial

**DOI:** 10.1007/s00520-022-07537-x

**Published:** 2022-12-19

**Authors:** Gunnhild Jakobsen, Karin Sjue, Ørnulf Paulsen, Stein Kaasa, Marianne Jensen Hjermstad, Pål Klepstad

**Affiliations:** 1 Department of Public Health and Nursing, Faculty of Medicine and Health Sciences, Norwegian University of Science and Technology (NTNU), and Cancer Clinic, St. Olavs hospital, Trondheim University Hospital, Trondheim, Norway; 2 Department of Oncology, Vestfold Hospital Trust, Tønsberg, Norway; 3 Institute of Clinical Medicine, European Palliative Care Research Centre, Department of Oncology, Oslo University Hospital, Oslo, Norway, University of Oslo, Oslo, Norway, and Palliative Care Unit, Telemark Hospital Trust, Skien, Norway; 4 European Palliative Care Research Centre (PRC), Department of Oncology, Oslo University Hospital, and Institute of Clinical Medicine, University of Oslo, Oslo, Norway; 5 Regional Advisory Unit in Palliative Care, Department of Oncology, Oslo University Hospital, Oslo, Norway, and European Palliative Care Research Centre, Department of Oncology, Oslo University Hospital, Oslo, Norway, and Institute of Clinical Medicine, University of Oslo, Oslo, Norway; 6 Department of Anaesthesiology and Intensive Care Medicine, St. Olavs Hospital, Trondheim University Hospital, Trondheim, Norway, and Department of Circulation and Medical Imaging, Faculty of Medicine and Health Sciences, Norwegian University of Science and Technology NTNU, Trondheim, Norway

**Keywords:** Advanced cancer, Insomnia, Sleep, Pharmacological treatment, Zopiclone, Randomized controlled trial

## Abstract

**Purpose:**

Insomnia is frequent in patients with advanced cancer, and a variety of pharmacological agents is used to treat this condition. Still, few clinical trials have investigated the effectiveness of pharmacological sleep therapies in this patient group. We aimed to study the short-term effectiveness of zopiclone on sleep quality in patients with advanced cancer who report insomnia.

**Methods:**

A randomized, double-blind, placebo-controlled, parallel-group, multicenter, phase IV clinical trial in adult patients with metastatic malignant disease and insomnia. Patients were treated with zopiclone or placebo for six subsequent nights. Primary end point was patient-reported sleep quality during the final study night (NRS 0–10). Secondary end points were patient-reported sleep onset latency (SOL) and total sleep time (TST).

**Results:**

Forty-one patients were randomized, with 18 being analyzed in the zopiclone group and 21 in the placebo group. Median age was 66, median Karnofsky performance score was 80, and 56% were male. Mean sleep quality at end of study was 2.9 (CI 2.3 to 3.8) in the zopiclone group and 4.5 (CI 3.6 to 5.4) in the placebo group (*p* = 0.021). At end of study, SOL was significantly different between the treatment groups: zopiclone 29 min (CI 13 to 51) and placebo 62 min (CI 40 to 87) (*p* = 0.045). TST was not significantly different across groups: zopiclone 449 min (403 to 496) and placebo 411 min (CI 380 to 440) (*p* = 0.167).

**Conclusion:**

Zopiclone improved short-term patient-reported sleep quality in this cohort of patients with advanced cancer.

**Trial registration:**

ClinicalTrials.gov Identifier: NCT02807922.

## Introduction

Sleep disturbances are frequent in patients with advanced cancer, with insomnia being the most prevalent sleep disorder [1]. The cancer disease and cancer treatments place patients at increased risk for disruption of normal behaviors and physiological states that are associated with restful sleep [2, 3]. Insomnia may occur at various points in the patient’s disease and treatment trajectory [4, 5].

Treatment for sleep disturbances in advanced cancer should address the multifactorial and manageable causes of sleep disturbances [6]. However, relief of symptoms such as pain, fatigue, anxiety, and depression does not necessarily lead to improvement of insomnia [2, 7]. Thus, in cancer patients as in other populations, sleep specific interventions must be considered. The recommended therapy for sleep disturbances is a stepwise approach starting with non-pharmacological intervention with cognitive behavior therapy for insomnia (CBT-I). If these are ineffective, pharmacological sleep interventions are a next step [6, 8]. A limitation for the implementation of CBT-I in cancer clinics is the lack of health professionals formally trained in these techniques. In addition, the number of CBT-I sessions, usually four to eight, might be too demanding for patients during cancer treatment. Thus, for patients with advanced cancer, pharmacological therapy is often used because of its rapid and relatively immediate effects [9, 10].

Several classes of sleep medications are used to treat sleep disturbances in cancer patients [11]. In a multicenter European study, the non-benzodiazepine hypnotic agent, zopiclone, was used by 348 of 2282 patients and was the most frequently used hypnotic agent in these patients with advanced cancer [11]. Zopiclone is one of the currently available therapies of insomnia. However, there is limited data from randomized controlled trials about the efficiency of hypnotic drugs in patients with advanced cancer, indicating a lack of evidence-based knowledge in the pharmacological treatment of insomnia in this group [2, 6, 8]. Patients with advanced cancer are in a different clinical situation than other patients with sleep disturbances, due to the limited life expectancy and the complexity of symptoms. Thus, findings from other patient populations cannot be extrapolated. In this group of patients, a well-designed randomized controlled trial is needed to determine the effectiveness of zopiclone on sleep quality to advise the clinical management of insomnia in palliative care populations.

On the basis of the lack of knowledge in pharmacological treatment of insomnia in patients with advanced cancer, we designed a double-blind, placebo-controlled, parallel-group RCT with the primary objective to evaluate the short-term effectiveness of zopiclone on self-reported sleep quality in patients with advanced metastatic cancer who report insomnia [12]. The secondary objectives were to study the effectiveness of zopiclone on mean self-reported sleep onset latency (SOL) and total sleep time (TST).

## Methods

The trial was a randomized, double-blind, placebo-controlled, parallel-group, phase IV clinical trial. Three palliative care and outpatient oncology services in Norway participated in the study: St. Olavs hospital, Trondheim University Hospital, Vestfold Hospital Trust, Tønsberg, and Telemark Hospital Trust, Skien.

### Participants

Eligible adult patients had verified malignant metastatic or disseminated disease (stage IV) and insomnia. Insomnia syndrome in the context of cancer was defined as self-reported difficulty initiating sleep (greater than 30 min to sleep onset) and/or difficulty maintaining sleep (greater than a 30-min nocturnal waking time) and/or waking up earlier than desired, for at least 3 nights per week. Furthermore, sleep problems should significantly impair daytime functioning, e.g., altered level of functioning, feeling tired, and lack of energy, as reported by the patients [13]. Main exclusion criteria were ongoing or previous treatment (within last 4 weeks) for more than 3 consecutive days with medications given for insomnia, adverse reactions to zopiclone, history of substance abuse, and concomitant use of rifampicin or erythromycin. Informed consent was signed before any study related procedures were done. Patients were randomized to either the zopiclone arm or the placebo arm using the web-based randomization system administered by the Clinical Research Unit, Faculty of Medicine and Health Sciences, Norwegian University of Science and Technology (NTNU)/St. Olavs hospital, Trondheim University Hospital.

### Intervention

Patients were randomized to zopiclone “Actavis” or placebo for 6 subsequent nights. The initial dose of zopiclone/placebo was 3.75 mg (dose level I), self-administered 30 min before bedtime, started after 1 night (baseline) with sleep quality assessment and symptom assessment. Patient-reported sleep quality was assessed by study personnel by phone after 2 and 4 nights on study medication, using a numerical rating scale (NRS) “Please circle the number that best describes how you feel now,” where 0 = best sleep, and 10 = worst possible sleep. If the NRS was ≥ 4 after 2 nights, the dose was increased to 5 mg (dose level II) zopiclone/placebo, and similarly if the NRS was still ≥ 4 after 4 nights, the dose was further increased 7.5 mg (dose level III) zopiclone/placebo. If patient-reported sleep quality was NRS < 4 after 2 or 4 nights, the patient would continue with the same dose. Dose titration was recorded in the WebCRF. Kragerø Tablettproduksjon AS, Norway produced, blinded, packed, and labeled identical looking and identical tasting zopiclone and placebo tablets. The study drug was blinded for patients, sites, and study personnel. The Unit for Applied Clinical Research, NTNU was external monitor for the trial.

### End points and assessments

The primary end point was patient-reported sleep quality during the final study night with treatment (night 6), assessed at daytime after night 6 using NRS 0–10, 0 = best sleep, 10 = worst possible sleep. Secondary end points were patient-reported sleep onset latency (SOL, i.e., how many minutes it takes to fall asleep starting from the moment of intention to fall asleep) and total sleep time (TST, i.e., the actual time slept in minutes) the final study night with treatment (night 6). All end points were reported in a sleep diary. Patients completed the sleep diary every morning when getting up. Patient-reported overall sleep quality, a predefined explorative end point, was assessed at baseline and at daytime after night 6 using the Pittsburgh Sleep Quality Index (PSQI) which in this study was modified to assess sleep quality during the past week [14]. Cancer-related symptoms (pain, drowsiness, tiredness, nausea, lack of appetite, shortness of breath, depression, anxiety, well-being, constipation, and vomiting) were measured using a NRS 0–10 from the European Association for Palliative Care (EAPC) basic dataset [15]. At the end of treatment, the patients were asked “How satisfied were you with the effect of the sleep medication”? with response categories: “not satisfied” (i.e., not at all satisfied, dissatisfied, and neither satisfied nor dissatisfied) or “satisfied” (i.e., satisfied and very satisfied) to assess their global perceived treatment benefits [16]. Performance status was rated by the Karnofsky performance status (KPS) [17], and sociodemographic and medical characteristics were obtained from the medical records by study personnel.

### Statistical analysis

The estimated sample size was 33 patients needed in each group, using a significance level of 5% and 80% power. The predefined minimal clinical important difference between the zopiclone and placebo group was 2 for the primary outcome sleep quality, scored on the NRS 0–10. Sample size calculations are presented in the protocol paper [12]. Demographic variables are reported as means with standard deviations (SD) or frequencies. In the comparison of sleep quality between the two groups after night 6 of using the study drug, independent Student’s *t*-test was used for continues variables. Additionally, a general linear model (ANCOVA) including baseline values of sleep quality as a covariate was used to assess differences in sleep quality at night 6 between the intervention group and the control group. The Pearsons Chi square test was used to compare the number of patients in each treatment group reporting satisfaction with study treatment. Intention-to-treat analyzes were used including all randomized patients regardless of protocol adherence. A two-sided *P* value ≤ 0.05 was considered statistically significant. Statistical analyses were performed using IBM SPSS Statistics for Windows, Version 27 Armonk, NY: IBM Corp.

## Results

### Baseline characteristics and cancer-related symptoms

**Fig. 1:**
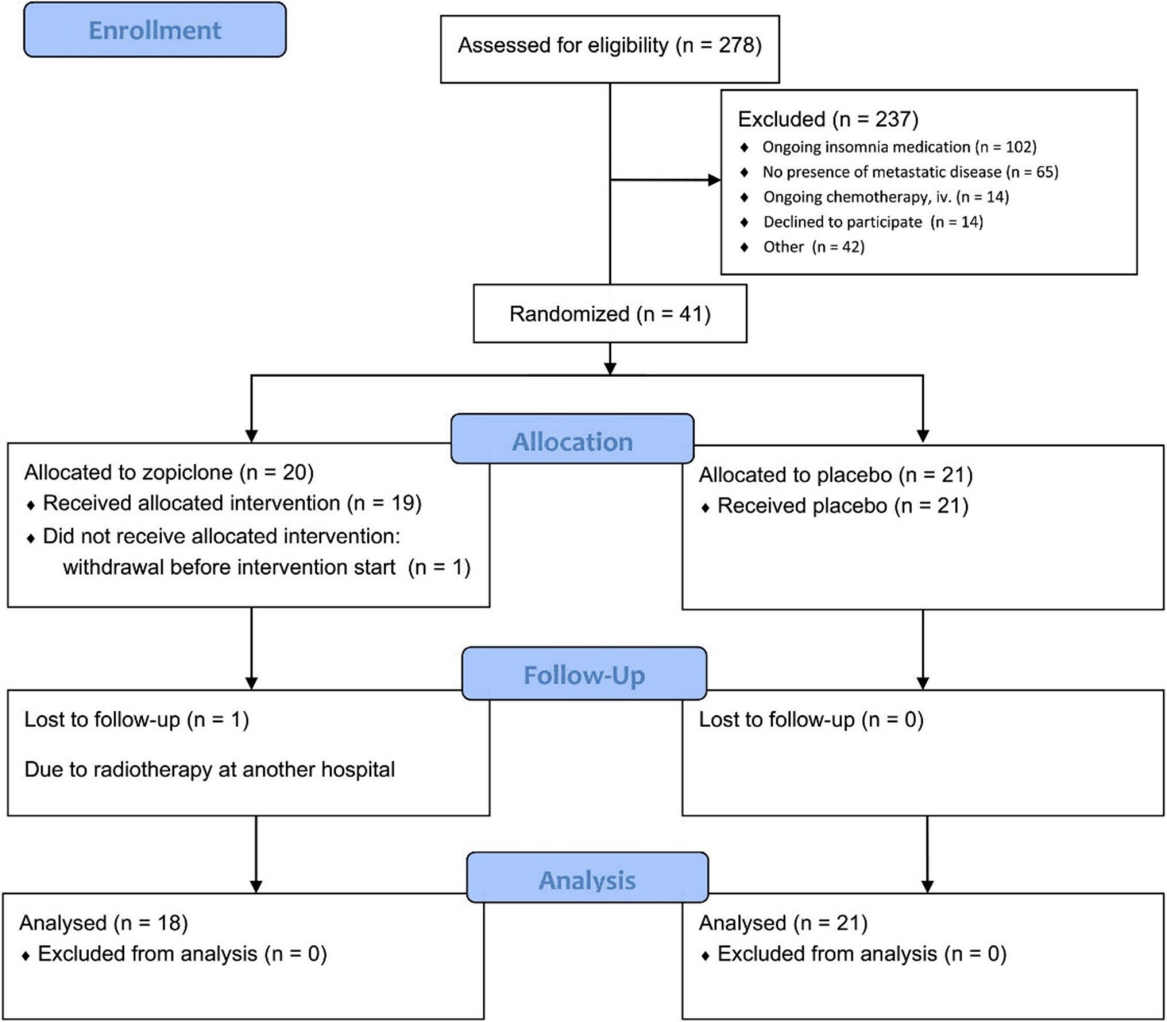
CONSORT diagram

From January 2017 to April 2020, a total of 278 patients were screened for eligibility. Main reasons for non-participation were ongoing treatment with medication given for insomnia (*n* = 102) and no presence of metastatic disease (*n* = 65), (Fig. 1). Forty-one patients were randomized to zopiclone or placebo; 20 were allocated to the zopiclone group and 21 to the placebo group, of which 18 could be evaluated in the zopiclone group and 21 in the placebo group (Fig. 1). Overall, median age was 66 years (range 26–79), median Karnofsky performance score was 80, and 56% of patients were male. Table 1 presents baseline demographic and clinical characteristics for each treatment group. The presence of cancer-related symptoms (mean scores, SD) at baseline and end of study is presented in Table 2.

**Table 1:** Demographic and clinical characteristics at baseline

Characteristic	Zopiclone (*n* = 18)	Placebo (*n* = 21)
Age, years, median (SD)	61 (14.3)	68 (12.4)
Sex, *n* (%)	55.6	57.1
Male	10 (56)	12 (57)
Female	8 (44)	9 (43)
Tumor type, *n* (%)		
Breast cancer	2 (11.1)	2 (9.5)
Gastrointestinal cancer	7 (38.9)	5 (23.8)
Hemotological cancer	4 (22.2)	4 (19.0)
Urological cancer	3 (16.7)	4 (19.0)
Malignant melanoma	2 (11.1)	4 (19.0)
Other	0	2 (19.0)
KPS, median (SD)	80 (7.3)	80 (15.6)
Concomitant disease present, *n* (%)	10 (55.6)	8 (38.1)
Cardiac	2 (11.1)	3 (14.3)
Vascular	6 (33.3)	0
Lung	1 (5.6)	1 (4.8)
Gastrointestinal	1 (5.6)	1 (4.8)
Kidney	0	1 (4.8)
Psychiatric	1 (5.6)	0
Musculoskeletal	1 (5.6)	2 (9.5)
Endocrine	2 (11.1)	0
Oral daily morphine equivalent dose (mg), median (SD; min–max)	48 (109; 16–220)	30 (20.7; 26–64)
Number of other medications, median (SD)	4 (3.07)	4 (3.26)
WHO Step III opioid for cancer pain^1^, *n* (%)	3 (16.7)	3 (14.3)
Antidepressants^2^, *n* (%)	0	1 (4.8)
Benzodiazepines^3^, *n* (%)	1 (5.6)	0
Cortocosteroids^4^, *n* (%)	3 (16.7)	1 (4.8)

*SD*, standard deviation; *KPS*, Karnofsky Performance Score using a 0 (dead) to 100 (i.e., normal activity) scale; ^1^Opioids: fentanyl, morphine, ozycodone. ^2^Antidepressants (Sarotex), ^3^benzodiazepines (Sobril), ^4^corticosteroids (Prednisolon, Dexamethason)

**Table 2:** Symptom characteristics at baseline and after six nights intervention (end of study)

Symptoms^1^	Zopiclone	Mean (SD)^2^	Placebo	Mean (SD)^2^
	Baseline	End of study	Baseline	End of study
Pain	1.5 (2.0)	0.8 (1.4)	1.6 (2.0)	0.7 (1.3)
Drowsiness	4.4 (2.8)	1.2 (1.9)	3.2 (2.0)	2.3 (2.2)
Tiredness	3.2 (2.0)	1.8 (1.8)	3.7 (2.5)	2.0 (2.4)
Nausea	0.4 (1.2)	0.1 (0.2)	0.4 (1.0)	0.0 (0.0)
Lack of appetite	0.5 (1.1)	0.1 (0.2)	2.4 (3.0)	0.6 (1.6)
Shortness of breath	0.8 (1.6)	0.6 (1.4)	1.3 (2.5)	0.4 (0.7)
Depression	1.4 (1.6)	0.7 (1.2)	1.4 (2.2)	1.1 (1.7)
Anxiety	1.2 (2.0)	0.6 (1.0)	1.1 (1.7)	0.9 (1.8)
Well-being	2.9 (2.2)	2.9 (1.5)	3.3 (2.2)	2.9 (1.8)
Constipation	1.0 (1.6)	0.4 (1.0)	1.1 (2.5)	0.0 (0.0)
Vomiting	0.4 (1.7)	0.1 (0.5)	0.0 (0.0)	0.0 (0.0)

^1^Cancer-related symptoms assessed on a 0–10 NRS at baseline and after six nights of intervention (zopiclone and placebo), scores: 0 = best, 10 = worst. ^2^Standard deviation

### Patient-reported sleep quality

**Fig. 2:**
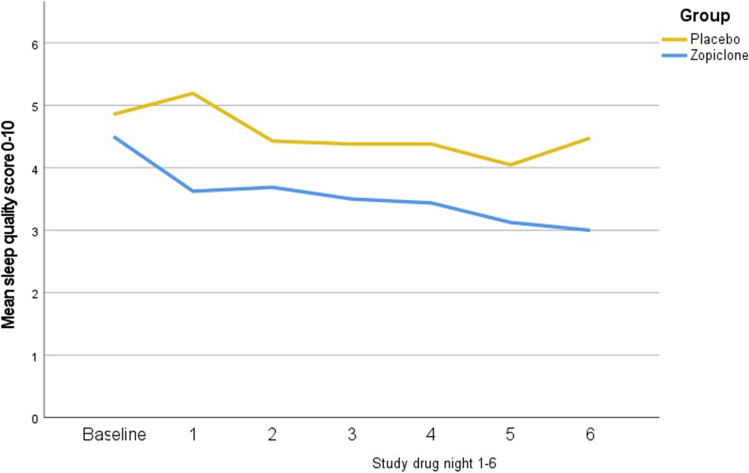
Profile of mean patient-reported sleep quality scores over time by treatment group

There were no baseline differences in mean patient-reported sleep quality (NRS 0–10) between the zopiclone group and the placebo group (4.9; 95%CI 4.2 to 5.6 vs. 5.4; 95%CI 4.5 to 6.0, *p* = 0.528). At night 6 with treatment, there was a statistically significant difference in sleep quality between the study groups (mean difference, 1.5; 95% CI, 0.3 to 2.8), with a mean NRS 2.9 (95% CI 2.3 to 3.8) in the zopiclone group and a mean NRS 4.5 (95% CI 3.6 to 5.4) in the placebo group (*p* = 0.021, Table 3). At night 6 with treatment, patient-reported SOL was 29 min (CI 13 to 51) in the zopiclone group and 62 min (CI 40 to 87) in the placebo group (*p* = 0.045), with a mean difference 33 min; 95% CI − 0.6 to 67.4). Total sleep time at night 6 with treatment was 449 min (CI 403 to 496) in the zopiclone group and 411 min in the placebo group (95% CI 380 to 440), (*p* = 0.167), with a mean difference − 38 min (95% CI − 93 to 17). Controlling for baseline scores on sleep (NRS 0–10) also showed significant differences in the sleep quality with zopiclone compared with placebo (ANCOVA, *F* = 5.64, *p* = 0.023) at night 6 with treatment. Figure 2 shows sleep quality (NRS 0–10) for baseline and study drug nights 1–6 by treatment group.

**Table 3:** Main results: primary outcome: patient-reported sleep quality (NRS 0–10). Secondary outcomes: patient-reported sleep onset latency (SOL) and total sleep time (TST)

Outcome	Zopiclone *n* = 18		Placebo *n* = 21		
	Mean	95% CI	Mean	95% CI	*P*^1^
Sleep quality, 0–10 NRS^2^	2.9	2.3 to 3.8	4.5	3.6 to 5.4	0.021
SOL, minutes^3^	29	13 to 51	62	40 to 87	0.045
TST, minutes^4^	449	403 to 496	411	380 to 440	0.167

^1^*P*-value from the *t*-test comparing differences in sleep quality night 6. ^2^Patient-reported sleep quality NRS 0–10, 0 = best sleep, 10 = worst possible sleep, ^3^sleep onset latency. ^4^Total sleep time

At baseline, the mean PSQI global score was 10.1 (CI 9 to11) in the zopiclone group and 9.4 (CI 8 to 11) in the placebo group. At end of the study, there were no significant difference in the global PSQI score between the groups with 8.13 (CI 6 to 10) in the zopiclone group vs. 9.7 (CI 6 to 10) in the placebo group (*p* = 0.179).

### Dose titration

All patients started with 3.75 mg (dose level I). In the zopiclone group, three patients (16.7%) continued this dose level, seven (38.9%) increased to dose level II (5 mg), and eight (44.4%) increased to 7.5 mg (dose level III) during the intervention. In the placebo group, one patient (4.8%) continued dose level I, eight (38.1%) increased to dose level II, and 12 (57.1%) increased to dose level III.

### Safety and satisfaction

In the zopiclone group, one patient reported temporary dizziness (reported at night 5 with treatment). In the placebo group, one patient reported transient headache (reported at night 4 with treatment). No serious adverse events was reported in either group. Patients’ global perceived treatment benefits assessed at the end of treatment showed that 62.5% in the zopiclone group were satisfied with the effect of the sleep medication (i.e., satisfied and very satisfied) vs. 15.4% in the placebo group (*p* = 0.027). In total, 15 (38.5%) reported “neither satisfied” nor “dis-satisfied” with the effect of the study drug.

## Discussion

This randomized, double-blinded, placebo-controlled, parallel-group, multicenter, clinical trial was designed to study the short-term effectiveness of zopiclone on patient-reported sleep quality in patients with advanced cancer who report insomnia. After six nights of intervention, patients receiving zopiclone reported significantly better sleep quality as compared with patients receiving placebo, adjusted for baseline sleep quality.

Two systematic reviews, one from 2013 and another from 2020, on the management of sleep disturbance in cancer identified no RCTs investigating pharmacological interventions for sleep disturbance in palliative cancer care [2, 8]. Nevertheless, a variety of pharmacological agents are in use to treat insomnia in patients with cancer [8]. Benzodiazepines, with zolpidem and trazodone as the most frequently used drugs in palliative care, are reported by patients to be helpful at the time of prescription [2, 18]. However, due to the absence of clinical trials with randomization and a placebo comparison group, the effectiveness of these drugs was previously unproven for use against insomnia in cancer patients [2, 18–21]. The present study is, to the best of our knowledge, the first RCT that presents the effectiveness of zopiclone in patients with advanced cancer.

Our results agree with trials which have demonstrated the effectiveness and safety of zopiclone in other populations [22–25]. A recent review including older adults with and without comorbidities demonstrated that zopiclone had positive effects on sleep quality leading to significant reduction in sleep latency, which corresponds with our results [23]. The eligibility criteria for this review were limited to older adults (≥ 60), which is comparable to the median age of 66 years in our trial. Another review including older adults with sleep disorders, with a mean age of 70 in all 24 trials, also demonstrated positive effects of zopiclone [26].

In addition to SOL and sleep quality, total sleep time is a commonly used outcome in studies of hypnotic drugs [23]. In the present trial, patients receiving zopiclone reported 38 min longer sleep duration as compared to patients receiving placebo. The difference between groups was not statistically significant, but might be clinically significant, as increased sleep duration between 23 and 29 min by the use of zopiclone is previously reported being effective to treat insomnia in older adults [23, 24]. On the other hand, an RCT comparing zopiclone, cognitive behavioral therapy, and placebo did not identify any difference in TST between the treatment arms [27]. Moreover, a meta-analysis demonstrated that several other hypnotic drugs such as doxepin, temazepam, eszopiclone, zolpidem, suvorexant, zaleplon, and triazolam yield significantly longer sleep duration than zopiclone, when compared to placebo [24].

Patients with advanced cancer represent a heterogeneous group of patients, differing in age, diagnoses, symptom burden, and expected survival time [15]. Regardless of these diversities, however, improving insomnia is an essential factor for optimizing of well-being and relief of symptoms in this group of patients. Cancer-related symptoms assessed on a 0–10 NRS at baseline and after six nights of intervention demonstrated a relative low intensity of pain, anxiety, and depression as compared to other studies in palliative care [18, 28–30]. As such, the findings from the present trial are applicable to patients with advanced cancer reporting mild cancer-related symptoms. The effectiveness of zopiclone on sleep quality in patients with higher symptom intensity might differ from the results from our trial.

To manage insomnia successfully, cognitive behavior therapy for insomnia (CBT-I) is considered the first-line treatment in adults of any age [31, 32]. This treatment incorporates cognitive and behavior-change techniques and targets dysfunctional attitudes, beliefs, and habits involving sleep [33]. It usually consists of psychoeducation/sleep hygiene, relaxation training, stimulus control therapy, sleep restriction therapy, and cognitive therapy [34]. In patients with cancer, CBT-I has been found to be associated with clinically and statistically significant improvements in patient-reported sleep outcomes [33]. However, given the lack of trained mental health professionals in palliative cancer care, the implementation of CBT-I is challenging. Besides, pharmacological interventions are often needed to provide immediate effective relief of symptoms, and when life expectancy is limited and patients experience complex symptoms such as pain and other problems, the goal of symptomatic relief overrides the concerns of long-term side effects [35]. Thus, for patients with advanced cancer, pharmacological therapy is often used as the first step after interventions directed at symptom control to remove the potential causative conditions for insomnia [18].

This trial has some major strengths. First, the trial is designed with methodological rigor, using a randomized, double-blind, placebo-controlled design, with patient recruitment at three centers. Second, the study included a titration protocol which recognized that patients need different doses. Third, the short intervention period of six nights with zopiclone or placebo, made it ethically feasible for patients to participate despite the blinding. Moreover, it kept the drop-out rate to a minimum, which is an important consideration in palliative care research [36].

The main limitation is the number of patients enrolled. Because of slow recruitment the trial was stopped before the predefined target of patients was reached. However, the risk for a potential type two error was mitigated by the statistically significant difference for the predefined primary end point. In fact, we believe that an interim analysis would have led to the trial being discontinued given the favorable outcomes. For this study, as in many other RCTs in palliative care studies, inclusion is difficult [37]. To improve palliative care, evidence-based knowledge on the effect of therapeutic interventions is needed, and measures should be taken to increase the feasibility of controlled trials also in this population [38–40]. Another limitation is that this study cannot report the effectiveness of long-term therapy with zopiclone. Nevertheless, study results showed positive effects of short-term use of zopiclone, indicating improved patient-reported sleep quality and reduced patient-reported sleep onset latency in patients with advanced cancer.

## Conclusion

In conclusion, this trial demonstrated in a randomized double-blind, placebo-controlled clinical trial that patient-reported sleep quality was significantly improved in cancer patients receiving short-term treatment with zopiclone compared to placebo. Future research should investigate the effectiveness of long-term treatment of zopiclone on sleep quality in patients with advanced cancer.

## Data Availability

The datasets generated and/or analyzed during the current study are available from the corresponding author on reasonable request.
